# Sharp wave-ripple complexes in a reduced model of the hippocampal CA3-CA1 network of the macaque monkey

**DOI:** 10.1186/1471-2202-16-S1-P15

**Published:** 2015-12-18

**Authors:** Juan F Ramirez-Villegas, Nikos K Logothetis, Michel Besserve

**Affiliations:** 1 Department of Physiology of Cognitive Processes, Max Planck Institute for Biological Cybernetics, Tübingen, 72076, Germany; 2 Graduate School of Neural & Behavioral Sciences, International Max Planck Research School, Eberhard-Karls University of Tübingen, Tübingen, 72074, Germany; 3 Centre for Imaging Sciences, Biomedical Imaging Institute, The University of Manchester, Manchester, M13 9PT, UK; 4 Department of Empirical Inference, Max Planck Institute for Intelligent Systems, Tübingen, 72076, Germany

## 

Sharp wave-ripple complexes observed in the hippocampal CA1 local field potential (LFP) are thought to play a major role in memory reactivation, transfer and consolidation. SPW-Rs are known to result from a complex interplay between local and upstream hippocampal ensembles. However, the key mechanisms that underlie these events remain partly unknown. In this work, we introduce a reduced, but realistic multi-compartmental model of the macaque monkey´s hippocampal CA3-CA1 network. The model consists of two semi-linear layers, each consisting of two-compartmental pyramidal neurons and one-compartmental perisomatic-targeting basket cells. Connections in the network were modeled as AMPA synapses, based on physiological and anatomical data. Notably, while auto-association fibers were prevalent in CA3, CA1 connectivity -inspired by recent findings- implemented a "feedback and reciprocal inhibition", dominated by recurrent inhibition and pyramidal cells-interneurons synapses. SPW-R episodes emerge spontaneously in the CA1 subfield LFP (which is assumed proportional to transmembrane currents across all compartments and medium resistivity): Episodes of short-lived high-frequency oscillations (ripples, 80-180 Hz) on top of a massive dendritic depolarization (< 20 Hz) with visual and quantitative characteristics observed experimentally [1]. Concomitantly, the CA3 subfield LFP presents episodes of quasi-synchronous neuronal bursting in the form of gamma episodes (25-75 Hz). The model reveals a lower bound for the minimal network that may generate SPW-R activity, and predicts a large number of features of in vivo hippocampal recordings in macaque monkeys [1]. Spike-LFP coherence analysis in CA1 displays reliable synchrony of spiking activity in the ripple LFP frequency band, suggesting that modeled SPW-R episodes reflect a genuine network oscillatory regime. Interestingly, interneuronal firing shows coherence increases concomitant with the beginning and the end of the SPW-R event, together with increases over gamma frequencies.

The model suggests that activity of both pyramidal neurons and interneurons is critical for the local genesis and dynamics of physiological SPW-R activity. Unlike other models, we found that it is interneuronal silence, not interneuronal firing that triggers these fast oscillatory events, in line with the fact that unbalanced excitability of selected pyramidal cells marks the beginning of single network episodes. Interneuronal silence quickly increases population firing of pyramidal cells. The interneuronal population activity increases with some latency due to the unbalanced excitatory drive, becoming pivotal to pyramidal cell activity, and further pacing pyramidal cells due to interneuronal fast kinetic properties. Our modeled data suggests that this effect is possibly mediated by a silencing-and-rebound-excitation mechanism, maintaining the frequency of the field oscillation bounded to the ripple range. The reduced model suggests a simple mechanism for the occurrence of SPW-Rs, in light of recent experimental evidence. We provide new insights into the dynamics of the hippocampal CA3-CA1 network during ripples, and the relation between neuronal circuits' activity at meso- and microscopic scales. Finally, our model exhibits characteristic cell type-specific activity that might be critical for the emergence of physiological SPW-R activity and therefore, for the formation of hippocampus-dependent memory representations.
